# Clinical and Epidemiological Features Among Patients with Wrist Arthroscopy Surgery: A Hospital‐based Study in China

**DOI:** 10.1111/os.12746

**Published:** 2020-07-29

**Authors:** Yao‐bin Yin, Bo Liu, Jing Zhu, Shan‐lin Chen

**Affiliations:** ^1^ Department of Hand Surgery Beijing Ji Shui Tan Hospital Beijing China

**Keywords:** Epidemiology, TFCC injury, Ulnar impaction syndrome, Ulnar‐sided wrist pain, Wrist arthroscopy

## Abstract

**Propose:**

To analyze the clinical and epidemiological features of patients who underwent wrist arthroscopy procedures.

**Methods:**

This is a cross‐sectional epidemiological study. The study included a cohort of patients who underwent wrist arthroscopy procedures in a national orthopaedic referral center from 1 February, 2014 to 1 February, 2019. The medical records, diagnosis, and every wrist arthroscopy procedure of all the patients were collected and reviewed. The epidemiological features, detail of the diagnoses, and the procedures of all the patients were systemically analyzed. All the patients were divided into two groups: trauma and non‐trauma conditions. The complexity of the wrist arthroscopy procedure was classified into simple procedures (exploration or debridement) and complex procedures (repair or reconstruction). χ^2^ test was used to compare proportions between the procedures of different complexity and the two groups of patients.

**Results:**

A total of 533 patients (332 males and 201 females) were included in this study. More than half (56%) of the patients were in the age group 21–40 and nearly two thirds (62%) of all the 533 patients were male. The diagnoses of all the patients could be classified into eight categories: (i) TFCC injury; (ii) ulnar impactions syndrome; (iii) carpal trauma (carpal bone fractures and/or carpal ligament injures); (iv) distal radius fractures; (v) carpal bone cyst or necrosis; (vi) ganglion cyst; (vii) wrist arthritis; and (viii) disorders of small joint of the hand. The most common conditions treated with wrist arthroscopy were TFCC injury (172 cases), followed by carpal trauma (125 cases) and ulnar impaction syndrome (84 cases). The simple arthroscopic procedures (exploration or debridement) account for 53% of all the procedures while complex reparative or reconstructive procedures account for 47%. There was a significant difference in the proportion between simple procedures and complex procedures in both trauma and non‐traumatic patients. Repair or reconstruction procedures were more frequently performed for wrist trauma patients, whereas exploration or debridement procedures were more frequently performed for non‐trauma patients.

**Conclusions:**

The largest group of patients who underwent wrist arthroscopy surgery are those who complained of ulnar‐sided wrist pain and the commonly conducted wrist arthroscopy procedures have evolved from simple exploration/debridement to the more complex repair or reconstruction procedures in China.

## Introduction

Arthroscopy, first described in 1918 in a cadaver knee joint and in 1962 successfully as an operative procedure^1^, has equipped the orthopaedic surgeon with an excellent procedure to diagnose and treat intra‐articular pathologies. After successful use in large joints, the technique has been progressively extended onto smaller sized joints such as the shoulder, hip, ankle, elbow and wrist. Wrist arthroscopy was first reported in 1979 for diagnostic purposes^2^. From the late 1980s through the 1990s, arthroscopy has become an important means in the armory of a hand surgeon, and wrist arthroscopy is now the so‐called golden standard for diagnosing intra‐articular lesions in the wrist. Wrist arthroscopy is currently the most accurate tool for the diagnosis of intra‐articular pathology of the wrist. Wrist arthroscopy has revolutionized the practice of orthopaedics, allowing the surgeon to examine intra‐articular abnormalities in depth under magnification and optimum lighting conditions^3^. Although initially used for only diagnostic purposes, wrist arthroscopy has now become the treatment of choice worldwide for many derangements of the wrist^4^, ^5^, ^6^, ^7^. Wrist arthroscopy has steadily grown from a predominantly diagnostic tool to a valuable adjunctive procedure in the treatment of myriad wrist disorders. The wide list of indications for wrist arthroscopy is continuously growing and includes basic treatment of soft tissue pathologies as synovitis, fibrosis, stiffness, ganglia, management of triangular fibrocartilage complex (TFCC) tears, scapholunate and lunotriquetral ligament injuries and removal of loose bodies^8^, ^9^, ^10^. Osseous procedures include partial bone resections in scaphotrapeziotrapezoid (STT) and ulnocarpal or ulnostyloid impaction syndrome^7^, ^11^. The method has also gained wider acceptance in more sophisticated procedures as assisting reduction of intra‐articular distal radius fractures^12^, or scaphoid fractures, and in posttraumatic sequelae^13^, ^14^. Arthroscopically assisted osteotomy in intra‐articular distal radius malunions, treatment of scaphoid nonunions and arthroscopic arthrolysis has been described^5^, ^15^. Arthroscopic decompression of the lunate for Kienböck's disease, arthroscopically assisted partial wrist fusions and arthroscopic proximal row carpectomy have been described^9^, ^16^.

The main advantage of wrist arthroscopy surgery is that it could directly explore wrist anatomy structure, especially for intercarpal ligaments, which could not be observed by open surgery. Surgeons could not observe the articular surface of the distal radius using a traditional palmar approach for distal radius fracture unless they cut the radiocarpal ligament. Wrist arthroscopy could observe these ligaments and articular surface clearly with minimal invasion^6^, ^17^. Secondly, wrist arthroscopy could treat some wrist diseases with less soft tissue dissection and more cosmetic scar than open surgery. For example, wrist arthroscopy surgery could resect wrist ganglion by small incision and explore the ligament injury accompanying the ganglion^18^, ^19^. Some traditional open procedures could be replaced by wrist arthroscopy procedures. For example, arthroscopic bone grafting for scaphoid nonunion could be performed in most of the cases, avoiding massive surgical trauma to the soft tissue^5^, ^13^. More complex conditions, such as perilunate dislocations, could be treated in a minimally invasive manner by arthroscopic‐assisted procedures as well^20^. Another example is the quite aggressive open partial or total wrist arthrodesis procedures, which now could also be performed arthroscopically^9^, ^21^. Thirdly, the use of wrist arthroscopy could aid the diagnosis of many complex wrist conditions^8^. For example, arthroscopic exploration could reveal the accompanying injury of distal radius fractures, such as TFCC and scapholunate interosseous ligament, which often require surgical intervention^22^, ^23^.

While wrist arthroscopy surgery was reported in Chinese literature as early as 1987^24^, the application was rather limited until the 2010s. Wrist arthroscopy surgery has a clear role in acute traumatic, sub‐acute post‐traumatic, as well as chronic conditions. However, the procedure has an associated learning curve and can be technically demanding. A thorough knowledge of wrist anatomy and the anatomic landmarks as well as careful and skilledsurgical technique are required to allow a safe and appropriate arthroscopic treatment of disorders in the wrist joint. Wrist arthroscopy has been becoming one of the most dramatically expanding fields in orthopaedic surgery in China for the last decade. More and more open procedures were gradually replaced by arthroscopy procedures in China for wrist trauma or other disorders. The number of wrist arthroscopy surgeries has increased significantly during the past 5 years, which is mainly due to the growth of domestic wrist arthroscopy surgeons and the development of wrist arthroscopy education in China. The categories of diseases were relatively extensive and the procedures not only included exploration or debridement, but also more complex repair and reconstruction surgery. Although it started later than in foreign countries, the gap is not large. However, to the best of our knowledge, there is no systemic study reported on the clinical and epidemiological features of patients who underwent wrist arthroscopy procedures. This current study analyzed the clinical and epidemiological features of the patients who underwent wrist arthroscopy procedures in the hospital where the authors worked. This hospital is one of the national orthopaedic referral centers in China, where wrist arthroscopy was widely used for wrist pathology. The features of different diseases treated by wrist arthroscopy and the procedures performed were explored in this study. What wrist disorders could be treated by wrist arthroscopy were revealed in this study.

## Method

This study was approved by the Ethics Committee at the hospital where the authors worked. All methods were carried out in accordance with relevant guidelines and regulations and informed consent was obtained from all subjects.

### *Patient Inclusion*

Inclusion criteria: (i) patients who underwent wrist arthroscopy procedures between 1 Febuary 2014 and 1 Febuary2019 in the national orthopaedic referral center the authors worked in; and (ii) patients whose observation indicators could be retrospectively analyzed. Exclusion criteria: (i) patient surgery records were not clear enough to access; and (ii) outpatients.

### *Recorded Indicators*

We reviewed the medical records, diagnosis, and every wrist arthroscopy procedure of all the patients. The epidemiological features, detail of the diagnoses, and the procedures of all the patients were systemically analyzed.

### *Age and Gender*

The patients were grouped by age into 0–20, 21–40, 41–60, and 61‐plus. The distribution of age and gender was analyzed.

### *Diagnoses of the Patients*

The diagnoses of all the patients could be classified into eight categories: (i) TFCC injury; (ii) ulnar impactions syndrome; (iii) carpal trauma (carpal bone fractures and/or carpal ligament injures); (iv) distal radius fractures; (v) carpal bone cyst or necrosis; (vi) ganglion cyst; (vii) wrist arthritis; and (viii) disorders of small joint of the hand. The proportion of each kind of disease was analyzed.

### *Information for each Disease Category*

The diagnosis and clinical features of each disease category were revealed and the wrist arthroscopy procedures for each disease category were analyzed.

### *Proportion between Simple Procedures and Complex Procedures in Both Trauma and Non‐Traumatic Patients*

All the patients were divided into two groups: trauma and non‐trauma conditions. According to the diagnosis of the patients, TFCC injury, carpal trauma (carpal bone fractures and/or carpal ligament injures), distal radius fractures and injury of small joint of the hand were classified into trauma group. Non‐trauma group included: ulnar impactions syndrome, carpal bone cyst or necrosis, ganglion cyst, wrist arthritis and osteoarthritis of small joint of the hand. The epidemiological features of these two kinds of diseases were analyzed.

The complexity of the wrist arthroscopy procedure was classified into simple procedures (exploration or debridement) and complex procedures (repair or reconstruction).

The proportion between simple procedures and complex procedures in both trauma and non‐traumatic patients were analyzed.

### *Statistical Analysis*

Descriptive data were presented as numbers. The number of each category disease and composition ratios were analyzed. The distribution of gender, trauma and non‐trauma patients in different age groups were accessed. χ^2^ test was used to compare proportions between the procedures of different complexity and the two groups of patients. The reported p values are two‐sided. A *P* value of <0.05 was considered to be significant. The analyses were done with SPSS (version 25 for Windows, Chicago, IL, USA).

## Result

### *Age and Gender*

**Fig. 1:**
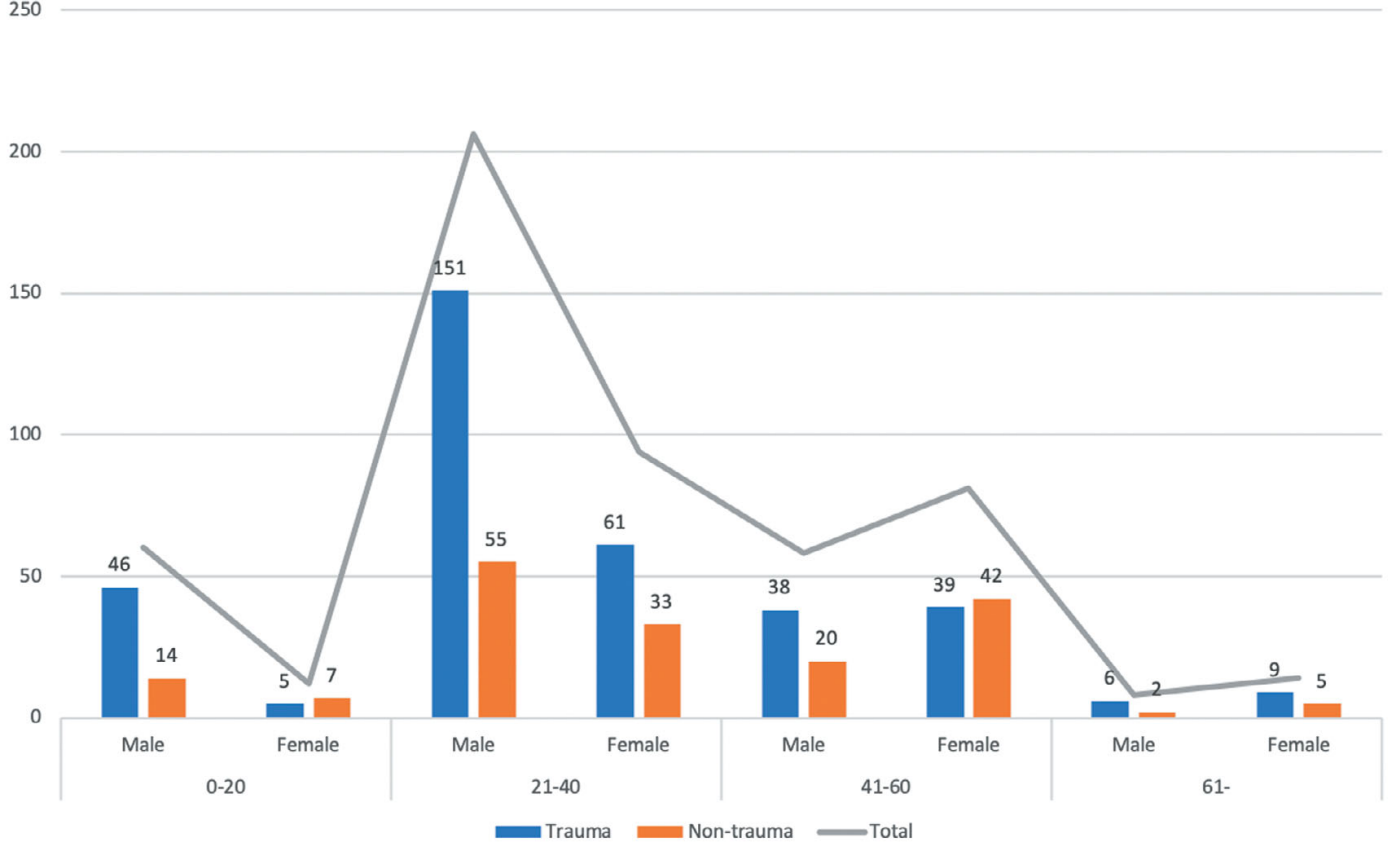
The age and gender distribution for trauma and non‐trauma diseases.

There were 533 (332 male and 201 female) patients included in this study. The demographic features were shown in Table 1. More than half (56%) of the patients were in the age group 21–40 and nearly two‐thirds (62%) of all the 533 patients were male. The details of age and gender distribution is shown in Fig. 1.

**TABLE 1:** Clinical and demographic features among patients treated with wrist arthroscopy surgery

Disease	Number (%)	Mean age (years)	Gender		Side	
			Male	Female	Left	Right
TFCC injury	167 (31.3%)	29	95	72	62	105
Carpal bone fractures and/or carpal ligament damage	123 (23.1%)	21	109	14	57	66
Ulna impaction syndrome	89 (16.7%)	37	43	46	34	55
Distal radius fractures	62 (11.6%)	44	34	28	34	28
Wrist arthritis	33 (6.2%)	38	17	16	9	24
Ganglion cyst	27 (5.1%)	33	16	11	12	15
Carpal bone cyst or necrosis	19 (3.6%)	33	14	5	7	12
Disorders of small joint of the hand	13 (2.4%)	48	4	9	4	9
Total	533 (100%)	31	332	201	219	314

### *Diagnoses of the Patients*

**Fig. 2:**
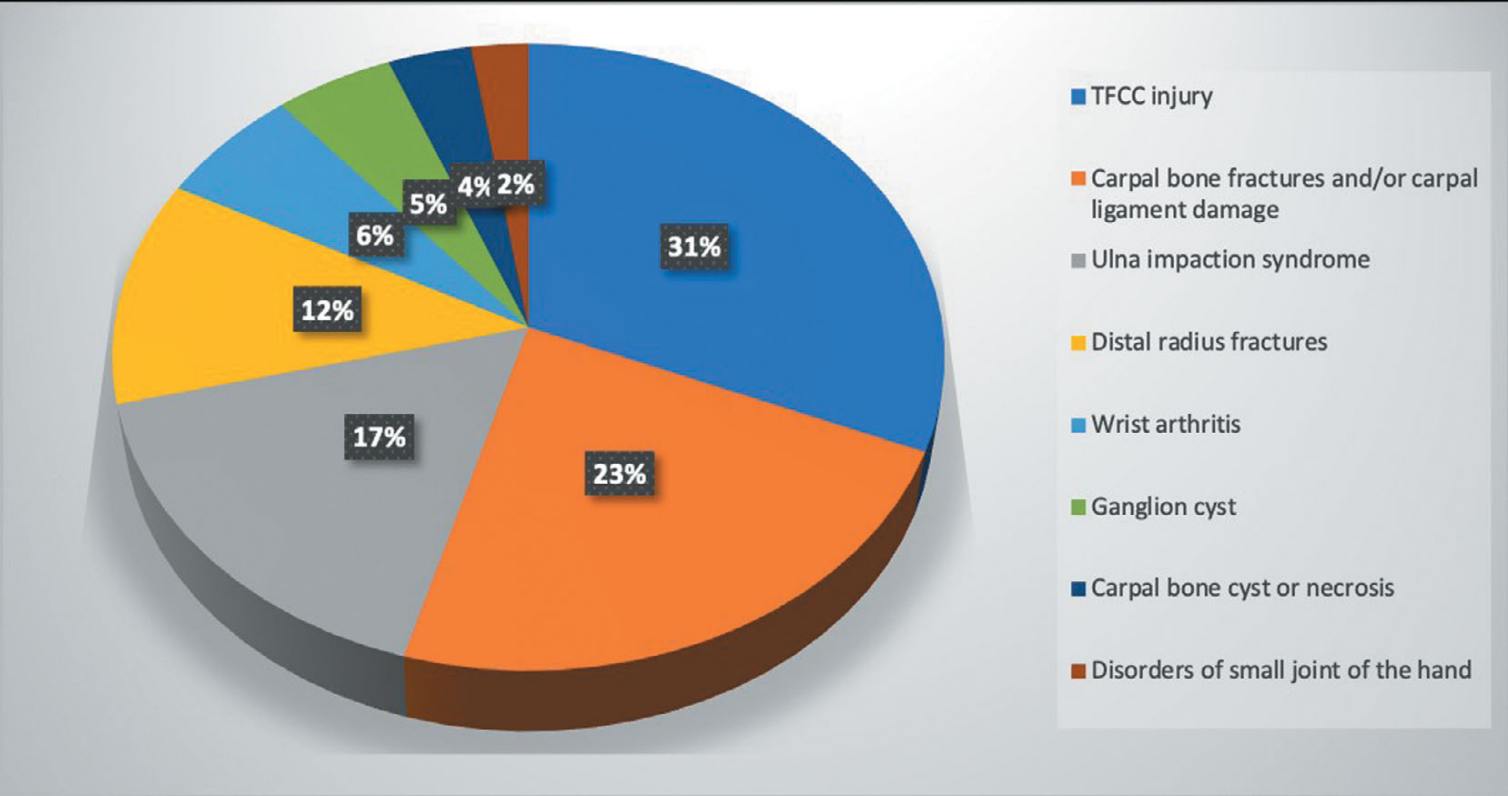
The proportion of various diseases.

The most common category was TFCC injury (167 cases, 31.3%), followed by carpal trauma (123 cases, 23.1%), and ulnar impaction syndrome (89 cases, 16.7%). The top three categories accounted for 71.1% of all the patients. Among the three categories, the TFCC injury and ulnar impactions syndrome are both characterized as pain over the ulnar side of the wrist. That means at least 48% of the arthroscopy procedures in this study were performed for the pathology located over the ulnar side of the wrist. The detail of the proportion of various conditions was showed in Fig. 2 and Table 1.

### *The Information for Each Disease Category*

#### *TFCC Injury*

One hundred and sixty‐seven traumatic TFCC injuries were treated with wrist arthroscopy procedures. The detail of the procedures for different types of TFCC injury were shown in Table 2.

**TABLE 2:** Detail of the treatment method for TFCC injury according to Palmer classification

Method	1a	1b	1c	1d	Dorsal tear
Repair	0	124	1	3	6
Debridement	3	9	0	4	0
Reconstruction	0	16	0	0	1
Total	3	149	1	7	7

#### *Carpal Trauma (Carpal Bone Fractures and/or Carpal Ligament Injuries)*

A total of 123 patients with carpal trauma underwent wrist arthroscopy procedures. The most common condition in this group was scaphoid fracture (82 cases, 66.7%). Wrist arthroscope was used to assist the percutaneous reduction and fixation of the acute scaphoid fracture. For sub‐acute scaphoid fracture or nonunion, arthroscopic assisted debridement and bone grafting was performed, which was done as an open surgery for many years before the introduction of modern wrist arthroscopic techniques. There were 21 patients with perilunate dislocation (with or without fracture) who were treated arthroscopically. The details of the procedures done for each condition in this category are shown in Table 3.

**TABLE 3:** Carpal bone fractures and (or) carpal ligament injury

Injury		Number
Scaphoid fracture	Acute	9
	Subacute	24
	Nonunion	49
Carpal ligament injury	Palmar radiocarpal ligament	2
	Scapholunate interosseous ligament	10
	Lunotriquetral interosseous ligament	3
Peri‐lunate dislocation (with or without fracture)		21
Scaphoid fracture combined with capitate fracture		1
Screw remove and debridement		4

#### *Ulnar Impaction Syndrome*

There were 89 patients diagnosed with ulnar impaction syndrome. Wrist arthroscopy was used to assess the degenerative changes of TFCC. The central disc attenuation or perforation was the most common lesion. Seventy‐three patients were treated with TFCC debridement arthroscopically followed by ulnar shortening osteotomy. Six patients underwent TFCC debridement only. Distal ulnar resection was performed for six patients because of combined distal radioulnar arthritis. Four patients underwent second‐look of TFCC arthroscopically at the time of implant removal.

#### *Distal Radius Fracture*

A total of 62 patients with distal radius fractures were treated with the assistance of wrist arthroscopy. Fifteen patients underwent arthroscopic debridement and arthrolysis at the time of implant removal. Among the remaining 47 cases, 31 were acute fractures (within 3 weeks); nine were sub‐acute fractures (from 3 weeks to 3 months); and seven were malunion fractures (over 3 months). The quality of reduction of articular surface was directly observed arthroscopically, which usually could not be clearly seen from the volar approach for distal radius fracture. The accompanying ligament injuries were identified and treated as required.

#### *Wrist Arthritis*

Among the 33 cases with wrist arthritis, 18 were rheumatoid arthritis, 13 were osteoarthritis, and two were gouty arthritis. Arthroscopic debridement and biopsy were performed for all rheumatoid arthritis and gouty arthritis patients. For the 13 osteoarthritis patients, arthroscopic debridement alone was performed for 10 patients, arthroscopic partial wrist arthrodesis was performed for two, and arthroscopic proximal row carpectomy for the remaining one.

#### *Ganglion Cyst*

The 27 wrist ganglion cysts were explored by wrist arthroscopy. One ganglion cyst was very close to the ulnar nerve and artery, and open surgery was performed. The remaining 26 cysts were resected arthroscopically. Among the 26 cases, 23 were located in the scapholunate joint, one located in ulnar dorsal of the wrist, one in ulnar palmar side of the wrist, and one in the area of scaphotrapeziotrapezoidal joint.

#### *Carpal Bone Cyst or Necrosis*

There were 14 cases of Kienböck disease and five cases of carpal bone cyst (two lunate, two triquetrum, and one scaphoid) that were treated arthroscopically. Thirteen cases of Kienböck disease were treated with arthroscopic‐assisted scaphoid‐capitate arthrodesis and one case was treated with arthroscopic debridement and open scaphoid‐capitate arthrodesis. All cases of carpal bone cysts were treated by arthroscopic debridement and bone grafting.

#### *Disorders of Small Joint of the Hand*

There were 13 cases of the first carpometacarpal joint disorders treated by wrist arthroscopy. Three cases with ligament laxity and subluxation of the first carpometacarpal joint were treated by arthroscopic debridement and thermal shrinkage. Ten cases with first carpometacarpal joint osteoarthritis were treated by arthroscopic distal trapezium osteotomy arthroplasty.

### *The Proportion between Simple Procedures and Complex Procedures in Both Trauma and Non‐Traumatic Patients*

The proportion of trauma and non‐trauma as well as simple procedures and complex procedures were analyzed. Among all the 533 cases, wrist trauma patients account for two‐thirds (355 cases) of all the patients, while one‐third (178 cases) were non‐trauma condition. The simple arthroscopic procedures (exploration or debridement) account for 53% of all the procedures while complex reparative or reconstructive procedures account for 47% (Table 4). There was a significant difference in the proportion between simple procedures and complex procedures in both trauma and non‐traumatic patients (χ^2^ test, *P* < 0.001). The simple arthroscopic procedures were used more frequently in non‐trauma patients, while the complex procedures were utilized more frequently in trauma patients (Table 4).

**TABLE 4:** Four fold table of arthroscopic procedures and trauma/non‐trauma patients (χ^2^ test)

	Procedures		
	Explore and/or debridement	Repair and/or reconstruction	Total	*P* value
Trauma	126	229	355	
Non‐trauma	156	22	178	
Total	282	251	533	<0.001

## Discussion

In this study, the top three conditions that underwent arthroscopic procedures were TFCC injury, carpal trauma, and ulnar impaction syndrome, which accounted for 71.1% of the total 533 cases. This result shows that the largest group of patients who underwent wrist arthroscopy surgery are those who complained of ulnar‐sided wrist pain, including the patients with TFCC injury and ulnar impaction syndrome^25^, ^26^.

Carpal trauma (carpal bone fractures and/or carpal ligament injuries) accounted for 23.1% of total cases. It has been reported that the procedures traditionally conducted by open surgery for the carpal trauma could be replaced by arthroscopic procedures^5^, ^13^. With the development of wrist arthroscopy techniques, more patients with carpal trauma will be treated with more minimally invasive arthroscopic procedures^20^.

Distal radius fracture is the most common fracture of upper extremity. In our hospital, it was usually treated by trauma surgeons, which might explain the relatively low proportion in this study conducted by hand surgeons. The promising role of wrist arthroscopy in the treatment of distal radius fracture has been reported recently^12^, ^17^.

For carpal bone cyst or necrosis, wrist ganglion, wrist arthritis, and disorders of small joint of the hand the use of wrist arthroscopy is also becoming more and more popular^4^, ^19^, ^21^.

The application of wrist arthroscopy was rather limited until the 2010s in China. Before that, wrist arthroscope was mainly used as a diagnostic tool. The procedures conducted in early days were mainly exploration or debridement. The data in this study has shown that a relatively great variety of the procedures were conducted in one national orthopaedic referral center. Not only simple procedures, like exploration or debridement, but also more complex reparative and reconstructive procedures were done frequently in the last 5 years. However, in a close look at the data of this study, wrist trauma patients account for two‐thirds (355 cases) of all the patients. Only one‐third of the patients were non‐trauma patients, and simple arthroscopic procedures were conducted more frequently for non‐trauma patients. One of the future directions of wrist arthroscopy for Chinese surgeons would be the development of more complex reparative or reconstructive surgical procedures for the non‐trauma patients.

One main limitation of this study is all the patient data was collected from one single hospital, which may not be representative of the whole Chinese population. However, the nature of this hospital, one of the largest national orthopaedic referral centers, is able to provide a sufficient number of patients and variety of procedures to adequately serve the purpose of this study.

In conclusion, the largest group of patients who underwent wrist arthroscopy surgery are those who complained of ulnar‐sided wrist pain. The top three conditions treated with wrist arthroscopy procedures were TFCC injury, carpal trauma, and ulnar impaction syndrome in this cohort of patients. Since the advent of wrist arthroscopy in China in the 1980s, the commonly conducted wrist arthroscopy procedures have gradually evolved from simple exploration/debridement to more advanced repair or reconstruction procedures.

## Declarations

### *Ethics Approval*

The ethical committee in Beijing Ji Shui Tan Hospital approved this study.

### *Consent to Participate*

All methods were carried out in accordance with relevant guidelines and regulations, and informed consent was obtained from all subjects.

### *Consent for Publication*

Not applicable.

### *Availability of Data and Material*

The datasets used and/or analyzed during the current study are available from the corresponding author on reasonable request

### *Authorship Declaration*

All authors listed met the authorship criteria according to the latest guidelines of the International Committee of Medical Journal Editors. All authors are in agreement with the manuscript.
